# Futile therapeutic nursing interventions in adult intensive care: A descriptive study

**DOI:** 10.1177/09697330241277988

**Published:** 2024-09-28

**Authors:** João Vítor Vieira, Henrique Oliveira, Sérgio Deodato, Felismina Mendes

**Affiliations:** 59207 Universidade Católica Portuguesa; Instituto de Telecomunicações; 59207 Universidade Católica Portuguesa; 70989 Universidade de Évora

**Keywords:** Critical care, intensive care, medical futility, nursing, nursing care

## Abstract

**Background:** Despite the progress made in recent decades on the phenomenon of futility in adult intensive care, recognizing it during clinical care practice remains a complex and sensitive process, during which questions are often raised for which concrete answers are difficult to find.

**Aims:** To analyze the frequency with which futile nursing interventions are implemented in critically ill patients admitted to adult intensive care in specific situations and how often futile autonomous and interdependent nursing interventions are implemented in the same population, as perceived by adult intensive care nurses.

**Research design:** Cross-sectional, quantitative, and descriptive study, which employed a questionnaire constructed specifically for this research to assess the perception of therapeutic futility in nursing in adult intensive care. Following an evaluation of the psychometric properties, the questionnaire was made available in an electronic format on the EUSurvey platform between August and October 2024. The data was analyzed between November 2023 and March 2024 using the statistical software packages SPSS and R.

**Participants and research context:** A simple random sample of nurses working in level II and level III intensive care units in Portugal.

**Ethical considerations:** Research ethical approvals were obtained, and the participants provided informed consent.

**Findings/results:** Four hundred and fourteen valid questionnaires were obtained. The results allow the identification of thirty-three statistically significant associations, the inference of intervals for the mean and median for the perception of futility of nursing interventions with a 95% confidence interval, and enable the hierarchization of nursing interventions implemented in critically ill patients admitted to adult intensive care units according to the nurses’ perception of their futility.

**Conclusion:** There is a balance in nurses' perception of the futility of their interventions in the specific situations analyzed. There is statistically significant evidence that interdependent nursing interventions are, in general, more frequently perceived as futile when compared to autonomous nursing interventions.

## Introduction

The high availability of resources in intensive care units (ICUs) enables the provision of care to critically ill patients experiencing highly complex situations. In addition to prolonging life, this can instigate the illusion that biological death can be avoided. Furthermore, it can lead to difficulty defining the prognosis and complex ethical dilemmas. It is also possible that the futility of interventions and treatments cannot be excluded.^1,2^

Despite the progress achieved in recent decades on the phenomenon of futility in intensive care, recognizing its presence throughout clinical practice remains a sensitive process. This is because questions are often raised that challenging to answer with certainty, since futility can be related to assumptions about the quality and quantity of life and can be invoked in ethically reprehensible ways.^3^

Nurses perform a professional practice that includes interventions that can be grouped into two categories: autonomous and interdependent. The first category of interventions is conducted by nurses in accordance with their professional qualifications and at their own discretion. The second comprises interventions prescribed by other professionals, with the nurse assuming responsibility for implementing them, ensuring continuity of care and evaluating outcomes.^4^

Since the 1980s, numerous authors have conducted research into therapeutic futility in intensive care. Nevertheless, the lack of knowledge about this issue in nursing, particularly regarding the futility of the interventions that nurses implement in these settings, appears to be significant. This may have ethical implications associated with the absence of benefit.^5^

It is an indisputable reality that inherent limitations constrain the provision of healthcare. Notwithstanding the systematic scientific development, it is fallacious to act as if science could triumph over all illnesses and death, even in intensive care. However, identifying when an intervention/treatment becomes futile and is no longer in the patient’s interest remains a highly challenging responsibility for health professionals.^6^ Furthermore, the futility phenomenon can exacerbate the financial burden associated with the provision of care, contribute to a greater scarcity of resources, and increase exhaustion, suffering, moral distress, and ethical conflicts among nurses. These factors can potentially impact the quality of care and professional relationships negatively.^7–9^

This cross-sectional, quantitative, and descriptive study, the largest of its kind conducted in Portugal exclusively with intensive care nurses on therapeutic futility, follows an extensive literature review on the central issue under analysis. It includes designing a questionnaire to assess the perception of therapeutic futility in nursing in adult intensive care. Following testing and validation, which confirmed good psychometric properties, the questionnaire was made available to all nurses from level II and III intensive care units in this country. The value of this study lies in its potential to contribute to the existing body of nursing knowledge regarding the futility of interventions implemented by nurses.

## Aims/research questions

This research analyzed the frequency with which futile nursing interventions are implemented in critically ill patients admitted to adult intensive care in specific situations and how often futile autonomous and interdependent nursing interventions are implemented in the same population, as perceived by adult intensive care nurses. To achieve this goal, the participants were presented with a conceptual proposal for therapeutic futility in nursing, constructed in a previous research stage according to a concept construction method. The proposal identifies two scenarios in which futile nursing interventions may be implemented. These are situations in which biophysiological indicators persistently contraindicate life, and situations of high irreversibility, which may be associated with a lack of benefit, transposition of acceptable limits, and excessively therapeutic measures. This is contingent upon the prevailing culture of intensive life-prolonging therapy and the level of criticality in which the patient finds himself. The proposed definition states that futile interventions can be autonomous or interdependent and associated with the performance of complementary medical diagnostic examinations or associated only with standards, protocols, routines, or scores.

In order to address the aforementioned objectives, the following research questions were formulated: (1) What is the frequency with which nurses perceive the implementation of futile nursing interventions in situations of persistence of biophysiological indicators incompatible with life and in situations of high irreversibility? (2) How frequently do nurses perceive the implementation of futile autonomous nursing interventions and futile interdependent nursing interventions?

In addition to the aforementioned objectives, the aim was to rank the interventions from those perceived as most futile to those perceived as least futile.

## Research design

### Ethical considerations and formal procedures

This study employs a cross-sectional, quantitative, and descriptive approach. The research was approved by the Health Ethics Committee of the “Universidade Católica Portuguesa” (reference CES-UCP n.^o^ 258). The data were collected through an anonymous questionnaire made available electronically for 90 days between August and October 2023 via the EUSurvey platform. The study and questionnaire were published by the “Ordem dos Enfermeiros” (the Portuguese Nurses Association) under the SAI-OE/2023/433 reference. Written informed consent was obtained from each participant at each stage of the study, from the construction of the questionnaire and evaluation of its psychometric properties to the application of the questionnaire.

### Designing the questionnaire

The initial phase of the research entailed designing the questionnaire. It was based on three main elements: (i) The literature review, which aimed to identify nursing assessment instruments, focused on the Nursing Activities Score^10^; (ii) the results of a qualitative research study conducted at an earlier stage, which sought to gather the nurses’ perceptions of therapeutic futility through a focus group comprising only expert nurses in adult intensive care, with a minimum of fifteen years of professional experience in these environments^11^; (iii) drafting the questionnaire’s final version, resorting to the Delphi technique to gather contributions from five participants, selected for convenience, all PhD nurses with thirty-six years of professional experience on average, who remained utterly anonymous. After receiving the experts’ responses, the questionnaire was subjected to further examination and revision. This process involved six resubmissions until all participants reached a consensus.^12,13^

Once completed, the questionnaire was submitted for evaluation by a sample of thirty level II and level III intensive care nurses to ascertain its clarity, rigor, and potential weaknesses and identify necessary changes.^13,14^ The nurses included in the sample were selected on a non-probabilistic convenience basis to ensure representation from levels II and III ICU settings, across all educational levels, professional categories, nursing specialties, unit types, and a range of professional experience.

### Statistical procedures

The statistical procedures conducted during this study were performed using the statistical software packages SPSS® (Statistical Package for the Social Sciences) version 29 and R version 4.3.2.

The fidelity assessment included assessing reliability and internal consistency.^12,13^ The test–retest method was employed to ensure reliability, while internal consistency was determined by calculating Cronbach’s alpha (α). At this stage, the validity assessment was limited to an evaluation of content validity.^12,13^

The final version (Table 1—Final Version of the Questionnaire) comprised nine questions about demographic and professional characteristics, which were classified as independent variables and represented various numerical, ordinal, dichotomous nominal, and polytomous nominal types. Additionally, twenty-two questions were distributed into three sections (one, two, and three), regarded as dependent variables with an ordinal scale response.

**Table 1.** Final version of the questionnaire.

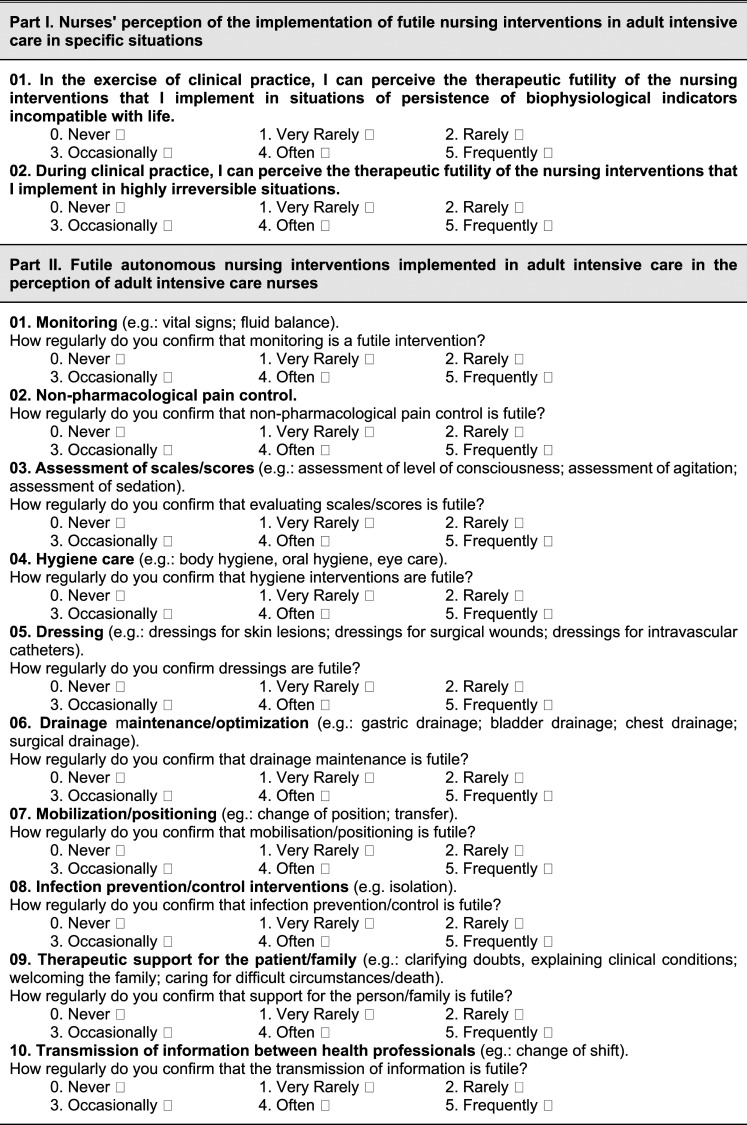
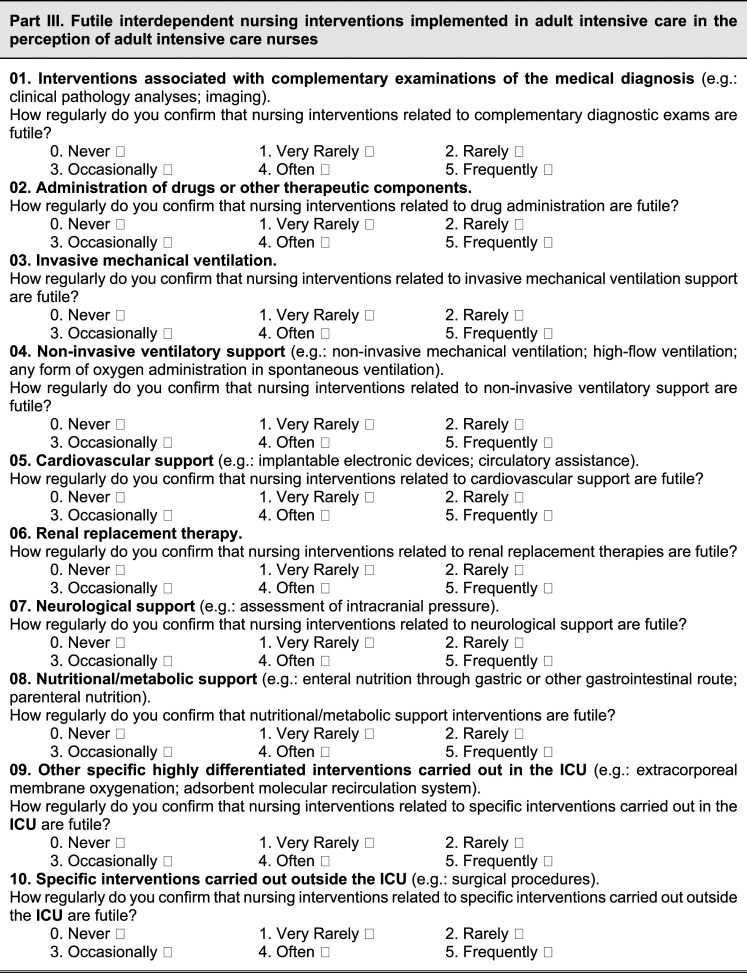

The first part comprised two inquiries on nurses’ perceptions of implementing futile nursing interventions in specific scenarios. The second and third sections comprised ten questions each, all about the nurses’ perceptions regarding the futility of autonomous and interdependent interventions. The responses to all questions in these sections are recorded on a 6-point Likert scale of frequency, with the following options: “Frequently,” “Often,” “Occasionally,” “Rarely,” “Very Rarely,” and “Never.” This Likert scale was selected for use in this study due to its prevalence in nursing and other health sciences research, wherein numerical values are assigned to each potential response. The six response options were selected to circumvent neutrality.^13^

The scale employs a rating system wherein the most negative response is assigned a value of “0,” while the most positive response is assigned a value of “5.” This implies that the minimum possible score for the perception of futility in the situations under analysis is 0 points and the maximum score is 10 points. Similarly, the minimum possible score for perceiving futility in autonomous and interdependent interventions is 0 points, while the maximum score is 100 points.

The second and third parts were subjected to the calculation of three scores. The first score was calculated for part two, which ranges from 0 (participants answer “0” for the 10 questions) to 50 points (participants answer “5” for the 10 questions), whose result is then multiplied by 2 to obtain a value between 0 and 100%. A similar procedure was carried out for part three. Ultimately, an overall score was also calculated (parts two and three), ranging from 0 (participants selected the answer “0” for all 20 questions) to 100 points (participants selected the answer “5” for all 20 questions).^13^

The study population consisted of nurses employed in level II or III adult ICUs in Portugal. Level II ICUs are distinguished by their capacity to facilitate invasive monitoring and maintain vital functions, although access to specific diagnostic resources and certain specialties may be constrained. These units admit patients requiring more detailed observation or intervention, including support for a failing single organ system, post-operative care, or transfer from a higher level of care. Level III ICUs represent the pinnacle of intensive care, offering specialized assistance in continuous intensive care, with access to advanced resources for the surveillance, monitoring, and therapy of critically ill patients. Patients admitted to these units require advanced respiratory support alone, essential respiratory support in conjunction with support from at least two organ systems, or support for multiorgan failure.

The probabilistic sampling method used in this study consisted of a simple random sample.^13^ The participants were monitored through the mandatory introduction of their professional ID number, which was concealed from the researchers. Each number was associated with a single questionnaire. Despite repeated attempts to obtain this information from several different sources, it was not possible to ascertain the exact number of level II and III intensive care nurses currently employed in Portugal. The sample was therefore calculated for a population of unknown size. Thus, for a 95% confidence interval with a margin of error of 5%, a minimum sample of 384.2 participants was considered.^15^

Statistical inference was calculated using the bootstrap confidence interval method. For this purpose, a sampling method of 1000 samples and a percentile confidence interval were employed to estimate the mean and median with a 95% confidence interval.^16,17^

## Findings/results

### Questionnaire validation

The questionnaire was administered on two occasions to the sample selected for the pre-test, with a two-week interval between each administration. In terms of reliability assessment (Table 2—Reliability Assessment), Spearman’s correlation coefficient (ρ) varied from ρ = 0.916 to ρ = 0.999 between the test and retest for twenty-one variables. Only one variable had a ρ-value lower than 0.900. These results demonstrate that the questionnaire exhibits high stability.^18,19^ The internal consistency, evaluated in the set of three parts containing the dependent variables of the questionnaire, was confirmed to be satisfactory, with Cronbach’s alpha (α) values ranging from α = 0.892 to α = 0.877 in the test and retest phases, respectively.^18^

**Table 2.** Reliability assessment.

Variable	ρ
Situations of persistence of biophysiological indicators incompatible with life (P01)	,955**
Highly irreversible situations (P02)	,941**
Monitoring (P03)	,999**
Non-pharmacological pain control (P04)	,991**
Use of scales/scores (P05)	,985**
Hygiene care (P06)	,916**
Dressing (P07)	,968**
Drainage maintenance/optimization (P08)	,971**
Mobilization/positioning (P09)	,938**
Infection prevention/control (P10)	,975**
Therapeutic support for the patient/family (P11)	,968**
Transmission of information between healthcare professionals (P12)	,980**
Interventions associated with complementary examinations of the medical diagnosis (P13)	,957**
Administration of drugs or other therapeutic components (P14)	,976**
Invasive mechanical ventilation (P15)	,994**
Non-invasive ventilatory support (P16)	,942**
Cardiovascular support (P17)	,943**
Renal replacement therapy (P18)	,976**
Neurological support (P19)	,917**
Nutritional/metabolic support (P20)	,927**
Other specific highly differentiated interventions carried out in the ICU (P21)	,717**
Specific interventions carried out outside the ICU (P22)	,946**

**Correlation is significant at the 0.01 level (2 tailed).

Content validity was ensured in the first phase by an extensive literature review and in the second phase by the judgement of experts selected for the expert panel. They conducted a thorough review, which allowed them to conclude that the questionnaire’s content was adequate to assess adult ICU nurses’ perceptions of therapeutic futility in nursing. A content validity index of over 0.90 was confirmed, indicating evidence of good content validity (Table 3—Content Validity Assessment).^12,13^

**Table 3.** Content validity assessment.

Variable	CVR	CVI
Situations of persistence of biophysiological indicators incompatible with life (P01)	1	0.927
Highly irreversible situations (P02)	1	
Monitoring (P03)	1	
Non-pharmacological pain control (P04)	1	
Use of scales/scores (P05)	1	
Hygiene care (P06)	1	
Dressing (P07)	0.6	
Drainage maintenance/optimization (P08)	0.6	
Mobilization/positioning (P09)	1	
Infection prevention/control (P10)	1	
Therapeutic support for the patient/family (P11)	0.6	
Transmission of information between healthcare professionals (P12)	1	
Interventions associated with complementary examinations of the medical diagnosis (P13)	1	
Administration of drugs or other therapeutic components (P14)	1	
Invasive mechanical ventilation (P15)	1	
Non-invasive ventilatory support (P16)	0.6	
Cardiovascular support (P17)	1	
Renal replacement therapy (P18)	1	
Neurological support (P19)	1	
Nutritional/metabolic support (P20)	1	
Other specific highly differentiated interventions carried out in the ICU (P21)	1	
Specific interventions carried out outside the ICU (P22)	1	

### Application of the questionnaire

The results were obtained from 414 valid questionnaires, corresponding to 265 female (64.01%) and 149 male (35.99%) participants. Only questionnaires with no missing data were considered valid. Concerning the participants’ sociodemographic and professional information (Table 4—Sample Characterization), it is noteworthy that the average *age* was 37.87 years (σ = 8.85 years), the average *length of professional experience* was 15.43 years (σ = 8.88 years), and the average *length of professional experience in adult intensive care* was 10.22 years (σ = 8.78 years). The participants’ *education level* exhibited considerable diversity, with 0.24% having obtained a bachelor’s degree (*n* = 1), 47.83% having pursued further studies at the graduate level (*n* = 198), 16.67% having completed a postgraduate degree (*n* = 69), 34.54% having obtained a master’s degree (*n* = 143), and 0.72% having acquired a doctorate (*n* = 3). Regarding the *nursing level*, the data indicate 54.11% of nurses (*n* = 224) and 45.89% of specialist nurses (*n* = 190), highlighting the specialties of Medical-Surgical Nursing – Critically Ill Patient (*n* = 62) and Rehabilitation Nursing (*n* = 54). Regarding the *ICU level*, 19.57% of the participants were drawn from level II units (*n* = 81), while 80.43% were from level III units (*n* = 333). The internal consistency assessment yielded an α = 0.924, indicating excellent internal consistency.^18^

**Table 4.** Characterization.

Sex	Female			Male	
	265 (64.01%)			149 (35.99%)	
Age	21–30	31–40	41–50	51–60	> 60
	102 (24.64%)	172 (41.55%)	97 (23.43%)	39 (9.42%)	4 (0.96%%)
	Mean: 37.87 anos; median: 37.00 anos; mode: 37 anos; standard deviation: 8.854 anos; minimum age: 22 anos; maximum age: 67 anos				
Education level	Bachelor’s degree	Graduate degree	Post-graduate degree	Master’s degree	Doctorate
	1 (0.24%)	198 (47.83%)	69 (16.67%)	143 (34.54%)	3 (0.72%)
Professional experience	< 1	1-4	5-10	11-20	> 20
	0 (0%)	39 (9.42%)	97 (23.43%)	174 (42.03%)	104 (25.12%)
	Mean: 15.43 anos; median: 14.00 anos; mode: 14 anos; standard deviation: 8.879 anos; minimum age: 1 ano; maximum age: 45 anos				
Professional experience in adult intensive care	< 1	1–4	5–10	11–20	> 20
	0 (0%)	128 (30.92%)	116 (28.02%)	122 (29.47%)	48 (11.59%)
	Mean: 10.22 anos; median: 8.00 anos; mode: 3 anos; standard deviation: 8.783 anos; minimum age: 1 ano; maximum age: 38 anos				
Nursing level	Nurse			Specialist nurse	
	224 (54.11%)			190 (45.89%)	
Nursing specialty	Community nursing: 7 (specialist nurse sample: 3.68%; total sample: 1.69%)				
	Medical-surgical nursing: 52 (specialist nurse sample: 27.36%; total sample: 12.56%)				
	Medical-surgical nursing—Critically ill patient: 62 (specialist nurse sample: 32.63%; total sample: 14.98%)				
	Medical-surgical nursing—Chronically ill patient: 3 (specialist nurse sample: 1.58%; total sample: 0.72%)				
	Rehabilitation nursing: 54 (specialist nurse sample: 28.42%; total sample: 13.04%)				
	Child health and pediatric nursing: 3 (specialist nurse sample: 1.58%; total sample: 0.72%)				
	Maternal health and obstetrics nursing: 2 (specialist nurse sample: 1.58%; total sample: 0.48%)				
	Mental health and psychiatric nursing: 7 (specialist nurse sample: 3.68%; total sample: 1.69%)				
ICU level	II			III	
	81 (19.57%)			333 (80.43%)	
ICU type	General/polyvalent				
	333 (80.43%)				
	Cardiology				
	20 (4.83%)				
	Cardiothoracic				
	28 (6.76%)				
	Surgical				
	2 (0.48%)				
	Neurosurgery				
	13 (3.14%)				
	Other				
	18 (4.35%)				

The frequency of responses to the different questionnaire items based on the 6-point Likert scale that was adopted for the study is presented in Table 5—Items Responses Frequency Distribution. The proportion obtained for each specific situation (P01 and P02) was calculated based on the sum of the responses (2945 points), and the proportion obtained for each nursing intervention (P03 to P22) was calculated based on the sum of the answers to the same questions (17520 points). This allowed for ranking both situations and interventions, from the most futile to the least. In terms of the situations under analysis, while some balance is evident, there is a greater perception of futility among nurses in *situations of high irreversibility* (1490 points) compared to situations of persistence of biophysiological indicators incompatible with life (1455 points). In terms of the perceived futility of nursing interventions, the score allows for the following hierarchy, from the most futile to the least: (1) *Interventions associated with complementary examinations of the medical diagnosis* (1348 points); (2) *Administration of drugs or other therapeutic components* (1193); (3) *Invasive mechanical ventilation* (1090); (4) *Renal replacement therapy* (1086); (5) *Specific interventions carried out outside the ICU* (1052); (6) *Monitoring* (1038); (7) *Cardiovascular support* (978); (8) *Neurological support* (966); (9) *Non-invasive ventilatory support* (962); 10) *Nutritional/metabolic support* (951); 11) *Use of scales/scores* (869); 12) *Drainage maintenance/optimization* (868); 13) *Dressings* (859); 14) *Hygiene care* (807); 15) *Other specific highly differentiated interventions carried out in the ICU* (763); 16) *Mobilization/positioning* (748); 17) *Infection prevention/control* (608); 18) *Non-pharmacological pain control* (595); 19) *Transmission of information between health professionals* (483); 20) *Therapeutic support for the patient/family* (256).

**Table 5.** Item response frequency distribution.

		Never (0)	Very rarely (1)	Rarely (2)	Occasionally (3)	Often (4)	Frequently (5)	Total score
Situations	P01	5 (1.21%)	12 (2.90%)	36 (8.70%)	134 (32.37%)	166 (40.10%)	61 (14.73%)	1455
	P02	4 (0.97%)	10 (2.42%)	32 (7.73%)	118 (28.50%)	188 (45.41%)	62 (14.98%)	1490
Autonomous interventions	P03	45 (10.87%)	74 (17.87%)	66 (15.94%)	117 (28.26%)	79 (19.08%)	33 (7.97%)	1038
	P04	146 (35.27%)	92 (22.22%)	83 (20.05%)	44 (10.63%)	40 (9.66%)	9 (2.17%)	595
	P05	66 (15.94%)	99 (23.91%)	69 (16.67%)	106 (25.60%)	56 (13.53%)	18 (4.35%)	869
	P06	80 (19.32%)	88 (21.26%)	90 (21.74%)	97 (23.43%)	47 (11.35%)	12 (2.90%)	807
	P07	59 (14.25%)	93 (22.46%)	97 (23.43%)	100 (24.15%)	53 (12.8%)	12 (2.9%)	859
	P08	49 (11.84%)	98 (23.67%)	106 (25.60%)	99 (23.91%)	49 (11.84%)	23 (5.56%)	868
	P09	80 (19.32%)	113 (27.29%)	86 (20.77%)	82 (19.81%)	48 (11.59%)	5 (1.21%)	748
	P10	142 (34.30%)	87 (21.01%)	94 (22.71%)	48 (11.59%)	26 (6.28%)	17 (4.11%)	608
	P11	268 (64.73%)	78 (18.84%)	38 (9.18%)	19 (4.59%)	10 (2.42%)	1 (0.24%)	256
	P12	192 (46.38%)	79 (19.08%)	63 (15.22%)	47 (11.35%)	28 (6.76%)	5 (1.21%)	483
Interdependent interventions	P13	14 (3.38%)	36 (8.70%)	46 (11.11%)	124 (29.95%)	122 (29.47%)	72 (17.39%)	1348
	P14	31 (7.49%)	48 (11.59%)	63 (15.22%)	112 (27.05%)	117 (28.26%)	43 (10.39%)	1193
	P15	37 (8.94%)	55 (13.29%)	80 (19.32%)	121 (29.23%)	93 (22.46%)	28 (6.76%)	1090
	P16	43 (10.39%)	82 (19.81%)	79 (19.08%)	134 (32.37%)	60 (14.49%)	16 (3.86%)	962
	P17	49 (11.84%)	81 (19.57%)	70 (16.91%)	121 (29.23%)	71 (17.15%)	22 (5.31%)	978
	P18	42 (10.14%)	56 (13.53%)	74 (17.87%)	124 (29.95%)	80 (19.32%)	38 (9.18%)	1086
	P19	61 (14.73%)	63 (15.22%)	74 (17.87%)	132 (31.88%)	61 (14.73%)	23 (5.56%)	966
	P20	52 (12.56%)	77 (18.60%)	89 (21.50%)	109 (26.33%)	66 (15.94%)	21 (5.07%)	951
	P21	122 (29.47%)	71 (17.15%)	57 (13.77%)	94 (22.71%)	54 (13.04%)	16 (3.86%)	763
	P22	28 (6.76%)	77 (18.60%)	73 (17.63%)	139 (33.57%)	73 (17.63%)	24 (5.80%)	1052

These results suggest that nurses may perceive interdependent interventions as more futile than autonomous interventions. The mean score for part two, which pertains to autonomous interventions, was 34.45 (±17.08), while the mean score for part three, which pertains to interdependent interventions, was 50.19 (±22.0). As the normality tests applied to both parts confirmed that neither follows a normal distribution (Table 6—Normality Tests), the Wilcoxon test was employed to ascertain whether there was a statistically significant difference between the means of parts two and three. The results of this test confirmed that there was indeed a statistically significant difference between the two means (Table 7—Wilcoxon Test).

**Table 6.** Normality tests.

	Kolmogorov**–**Smirnov^a^			Shapiro**–**Wilk		
	Statistic	df	Sig	Statistic	Df	Sig
Normality tests for variables						
Sex	,413	414	<,001	,607	414	<,001
Age	,074	414	<,001	,961	414	<,001
Education level	,310	414	<,001	,751	414	<,001
Professional experience	,077	414	<,001	,954	414	<,001
Professional experience in adult intensive care	,158	414	<,001	,910	414	<,001
Professional category	,362	414	<,001	,634	190	<,001
Nursing specialty	,305	190	<,001	,844	190	<,001
UCI level	,493	414	<,001	,485	414	<,001
UCI type	,461	414	<,001	,460	414	<,001
Situations of persistence of biophysiological indicators incompatible with life	,231	414	<,001	,879	414	<,001
Highly irreversible situations	,263	414	<,001	,864	414	<,001
Monitoring	,185	414	<,001	,927	414	<,001
Non-pharmacological pain control	,197	414	<,001	,860	414	<,001
Use of scales/scores	,177	414	<,001	,921	414	<,001
Performing hygiene	,158	414	<,001	,920	414	<,001
Dressing	,155	414	<,001	,929	414	<,001
Drainage maintenance/optimization	,155	414	<,001	,932	414	<,001
Mobilization/positioning	,193	414	<,001	,911	414	<,001
Infection prevention/control	,191	414	<,001	,865	414	<,001
Therapeutic support for the patient/family	,376	414	<,001	,660	414	<,001
Transmission of information between healthcare professionals	,268	414	<,001	,807	414	<,001
Interventions associated with complementary examinations of the medical diagnosis	,190	414	<,001	,904	414	<,001
Administration of drugs or other therapeutic components	,191	414	<,001	,915	414	<,001
Invasive mechanical ventilation	,190	414	<,001	,927	414	<,001
Non-invasive ventilatory support	,202	414	<,001	,927	414	<,001
Cardiovascular support	,191	414	<,001	,926	414	<,001
Renal replacement therapy	,188	414	<,001	,928	414	<,001
Neurological support	,201	414	<,001	,921	414	<,001
Nutritional/metabolic support	,166	414	<,001	,932	414	<,001
Other specific highly differentiated interventions carried out in the ICU	,176	414	<,001	,884	414	<,001
Specific interventions carried out outside the ICU	,206	414	<,001	,929	414	<,001
Normality tests for parts II and III						
Part two (P03 to P12)	,060	414	,001	,988	414	,002
Part three (P13 to P22)	,069	414	,000	,985	414	,000

^a^Lilliefors significance correlation.

**Table 7.** Wilcoxon test.

		*N*	Mean rank	Sum of ranks
Wilcoxon signed ranks test				
Part three	Negative ranks	71^a^	114.36	8119.50
Part two	Positive ranks	325^b^	216.88	70486.50
	Ties	18^c^		
	Total	414		

	Part_Three-Part_Two
Test Statistics^d^	
Z	−13.687^e^
Asymp. Sig. (2-Tailed)	.000

^a^Part_Three < Part_Two.

^b^Part_Three > Part_Two.

^c^Part_Three = Part_Two.

^d^Wilcoxon Signed Ranks Test.

^e^Based on negative ranks.

### Bivariate statistical analysis

The results of the Kolmogorov–Smirnov and Shapiro–Wilk normality tests indicate that the sociodemographic and professional characterization variables, as well as all the dependent variables in parts one, two, and three, do not follow a normal distribution at a statistical significance level of 5% (Table 6—Normality Tests). This finding, in conjunction with the nature of the variables, underscores the necessity for the utilization of non-parametric tests to ascertain the interrelationships between independent and dependent variables, as delineated by the following: The Spearman correlation coefficient (ρ) to evaluate the association between numerical and ordinal variables, as well as the association between two ordinal variables; the point-biserial correlation coefficient (r_pb_) to evaluate the association between dichotomous and ordinal variables; the Cramer’s V contingency coefficient (𝜙c) to evaluate the association between polytomous and ordinal variables.^18,19^

A bivariate statistical analysis examined the potential associations between sociodemographic and professional variables and the dependent variables. The analysis identified 33 statistically significant associations: seven negative associations with the variable *sex* (*sex* and *monitoring* r_pb_ = −0.098*^1^; *sex* and *use of scales/scores* r_pb_ = −0.176**^2^; *sex and drainage maintenance/optimization* r_pb_ = −0.118*; *sex* and *infection prevention/control interventions* r_pb_ = −0.184**; *sex* and *transmission of information between healthcare professionals* r_pb_ = −0.178**; *sex* and *invasive mechanical ventilation* r_pb_ = −0.112*; *sex* and *non-invasive ventilatory support* r_pb_ = −0.128**); five negative associations with variable *age* (*age* and *situations of persistence of biophysiological indicators incompatible with life* ρ = −0.139**; *age* and *highly irreversible situations* ρ = −0.100*; *age* and *therapeutic support for the patient/family* ρ = −0.112*; *age* and *nutritional/metabolic support* ρ = −0.120*; *age* and *specific interventions performed outside the ICU* ρ = −0.138**); seven positive associations with variable *education level* (*education level* and *monitoring* ρ = 0.220**; *education level* and *dressings* ρ = 0.156**; *education level* and *drainage maintenance/optimization* ρ = 0.113*; *education level* and *interventions associated with complementary examinations of the medical diagnosis* ρ = 0.156**; *education level* and *administration of drugs or other therapeutic components* ρ = 0.127**; *education level* and *cardiovascular support* ρ = 0.114*; *education level* and *other specific highly differentiated interventions carried out in the ICU* ρ = 0.111*); one negative and one positive associations with variable *professional experience* (*professional experience* and *situations of persistence of biophysiological indicators incompatible with life* ρ = −0.129**; *professional experience* and *specific interventions carried out outside the ICU* ρ = 0.105*); one positive association with variable *professional experience in adult intensive care* (*professional experience in ICU* and *mobilization/positioning* ρ = 0.098*); three positive associations with variable *professional category* (*professional category* and *monitoring* ρ = 0.165**; *professional category* and *hygiene care* ρ = 0.119*; *professional category* and *dressings* ρ = 0.157**); two associations with variable *nursing specialty* (*nursing specialty* and *situations of persistence of biophysiological indicators incompatible with life* *Φ*c = 0.202; *nursing specialty* and *highly irreversible situations* *Φ*c = 0.202); four positive associations with variable *ICU level* (*ICU level* and *highly irreversible situations* ρ = 0.142**; *ICU level* and *administration of drugs or other therapeutic components* ρ = 0.121*; *ICU level* and *renal replacement therapy* ρ = 0.137**; *ICU level* and *neurological support* ρ = 0.099*); two associations with variable *type of ICU* (*type of ICU* and *situations of persistence of biophysiological indicators incompatible with life* *Φ*c = 0.149; *type of ICU* and *highly irreversible situations* *Φ*c = 0.193).

### Statistical inference

The bootstrap confidence interval method allowed the calculation of statistics for the mean and median for the two situations and the twenty nursing interventions under study with a 95% confidence interval (Table 8—Statistical Inference by Bootstrap Confidence Interval).^16,17^

**Table 8.** Statistical inference by bootstrap confidence interval.

	Mean	Standard error	Confidence interval 95%		Median	Standard error	Confidence interval 95%	
			Inferior	Superior			Inferior	Superior
Situations of persistence of biophysiological indicators incompatible with life	3.51	0.05	3.42	3.62	4	0.14	4	4
Highly irreversible situations	3.6	0.05	3.50	3.69	4	0	4	4
Monitoring	2.51	0.07	2.37	2.65	3	0.12	3	3
Non-pharmacological pain control	1.44	0.07	1.30	1.57	1	0.06	1	1
Use of scales/scores	2.1	0.07	1.96	2.23	2	0	2	2
Hygiene care	1.95	0.07	1.82	2.09	2	0	2	2
Dressing	2.07	0.07	1.94	2.21	2	0	2	2
Drainage maintenance/optimization	2.1	0.06	1.98	2.22	2	0	2	2
Mobilization/positioning	1.81	0.07	1.67	1.94	2	0.28	1	2
Infection prevention/control	1.47	0.07	1.34	1.60	1	0.1	1	1
Therapeutic support for the patient/family	0.62	0.05	0.51	0.72	0	0	0	0
Transmission of information between healthcare professionals	1.17	0.06	1.05	1.30	1	0.24	0	1
Interventions associated with complementary examinations of the medical diagnosis	3.26	0.06	3.13	3.38	3	0.27	3	4
Administration of drugs or other therapeutic components	2.88	0.07	2.74	3.02	3	0	3	3
Invasive mechanical ventilation	2.63	0.07	2.51	2.76	3	0	3	3
Non-invasive ventilatory support	2.32	0.06	2.20	2.45	3	0.47	2	3
Cardiovascular support	2.36	0.07	2.23	2.5	3	0.42	2	3
Renal replacement therapy	2.62	0.07	2.48	2.76	3	0	3	3
Neurological support	2.33	0.07	2.19	2.47	3	0.39	2	3
Nutritional/metabolic support	2.3	0.07	2.16	2.43	2	0.34	2	3
Other specific highly differentiated interventions carried out in the ICU	1.84	0.08	1.70	1.99	2	0.26	1	2
Specific interventions carried out outside the ICU	2.54	0.07	2.41	2.67	3	0.07	3	3

For the mean, intervals with narrow amplitudes were confirmed for the two variables related to specific situations, namely, 0.19 for the *situation of high irreversibility* (3.42 to 3.62) and 0.20 for the *situation persistence of biophysiological indicators incompatible with life* (3.50 to 3.69). Concerning autonomous interventions, the ranges observed vary between 0.21 for *therapeutic support for the patient/family* (0.51 to 0.72) and 0.28 for *monitoring* (2.37 to 2.65). In the case of interdependent interventions, the intervals in question vary between 0.25, for *interventions associated with complementary examinations of the medical diagnosis* (3.13 to 3.38), and 0.29, related to *other specific highly differentiated interventions carried out in the ICU* (1.70 to 1.99).

The median’s lower and upper confidence intervals are confirmed to be equal to the median calculated for the two specific situations, 4. The lower and upper confidence intervals for autonomous interventions are equal to the median in eight interventions, except for *mobilization* and *positioning interventions* (1 to 2) and *transmission of information between health professionals* (0 to 1). The minimum and maximum limits of the confidence intervals for interdependent interventions are confirmed to be equal to the median in four instances, except for *interventions associated with complementary examinations of the medical diagnosis* (3 to 4), *non-invasive ventilatory support* (2 to 3), *cardiovascular support* (2 to 3), *neurological support* (2 to 3), *nutritional/metabolic support* (2 to 3), and *other specific highly differentiated interventions carried out in the ICU* (1 to 2).

## Discussion

This study, the first carried out in Portugal on therapeutic futility exclusively with ICU nurses, included a sample with socio-demographic and professional characteristics that support robust results regarding nurses’ perceptions of the phenomenon under study, highlighting the average *length of professional experience* of more than 15 years, the average *length of professional experience in adult ICU* of more than 10 years, and the significant proportions of nurses with postgraduate studies and specialization in nursing of more than 35% and 45%, respectively. In another study conducted in Portugal on medical futility, which involved health professionals and aimed to understand the meaning of futility and the reality of the end-of-life decision-making process in adult ICUs, nurses had a similar mean *age* of 38 years and reported an identical length of *professional experience* of 15 years, but a slightly shorter length of *professional experience in ICU* of 9 years.^20^

The results allow us to recognize that nurses perceive the implementation of futile interventions for critically ill patients admitted to the ICU. Of the situations studied, more than 88% of nurses perceive the implementation of futile interventions in *situations of high irreversibility* occasionally, often or frequently, and more than 87% of nurses perceive the implementation of futile interventions in *situations of persistence of biophysiological indicators incompatible with life* occasionally, often or frequently. Studies by Mohammadi and Roshanzadeh (2015), Rostami et al. (2019), and Moaddaby et al. (2021) obtained results consistent with the results of this study, as all the authors concluded that the majority of nurses have a moderate perception of the implementation of futile care in patients admitted to intensive care.^21–23^

The responses to the interventions were analyzed into two groups: autonomous and interdependent. The results show that nurses’ perceptions of futility were more pronounced in interdependent than autonomous interventions.

Concerning interdependent interventions, the results obtained are in line with the results of Yekefallah et al. (2015), who found that diagnostic procedures and drug administration were among the most frequent categories of futile interventions in intensive care,^24^ or those of Vieira et al. (2022), in which nurses unanimously identified the futility of some interdependent interventions, such as the excessive introduction of invasive devices or the administration of some drugs, such as vasoactive amines, despite refractory situations.^11^ In another study by Damps et al (2022), the authors reported in detail the interdependent nursing interventions that should be limited in situations of declared futility due to their intrinsic futility, highlighting intubation and mechanical ventilation (44.21%), mechanical and pharmacological support of the circulatory system (61.05%), extracorporeal membrane oxygenation (62.10%), renal replacement therapy (58.42%), transfusion of blood products (53.16%), and antibiotic therapy (40.53%).^25^ The interventions described by these authors are consistent with those identified in our study. Some are even at the top of the hierarchy of futile interventions.

A recent study by Choi et al. (2023) also presents conclusions consistent with our study’s results on interdependent interventions.^26^ These authors report that interventions that may be solely life-sustaining, such as administering drugs or other therapeutic components, invasive mechanical ventilation, and renal replacement therapy, are at the top of the hierarchy of interventions perceived as most futile in the care of critically ill patients. In the hierarchy of perceived futility proposed by our study, these interventions are ranked second, third, and fourth, respectively.^26^

The perceived futility of interventions shows some significant results for some autonomous interventions, such as *monitoring*, *use of scales/scores*, *drainage maintenance/optimization*, *dressing,* or *hygiene care*. These results are aligned with those obtained by Vieira et al. (2022), who identified a consensus in recognizing some futile autonomous nursing interventions, such as monitoring and nutrition exclusively for life prolongation, or interventions defined by routine, such as the evaluation of some scores.^11^ Although without identifying specific interventions, some authors confirm that a significant percentage of nurses identify a moderate to high level in the perception of the frequency of futile care,^27–29^ and there is even evidence to suggest that nurses have a higher perception of futility than other professionals.^30^

The bivariate statistical analysis allowed the identification of some statistically significant associations that were considered relevant.

In terms of associations between *sex* and nursing interventions, results suggest an association between men and perception of futility of *monitoring* (rpb = −0.098), *use of scales/scores* (rpb = −0.176), *drainage maintenance/optimization* (rpb = −0.118), *infection prevention/control* (rpb = −0.184), *transmission of information between healthcare professionals* (rpb = −0.178), *invasive mechanical ventilation* (rpb = −0.112), and *non-invasive ventilatory support* (rpb = −0.128). The five associations identified between *age* and specific situations and nursing interventions suggest that with increasing *age*, the perception of futility decreases in *situations of persistence of biophysiological indicators incompatible with life* (ρ = −0.139), in *situations of high irreversibility* (ρ = −0.100), in perception of futility of *therapeutic support for the patient/family* (ρ = −0.112), in *nutritional/metabolic support* (ρ = −0.120), in *specific interventions carried out outside the ICU* (ρ = −0.138), and vice versa.

Regarding education level, seven statistically significant positive linear associations were identified, indicating that as nurses’ education level increases, the perception of the futility of monitoring (ρ = 0.220), dressing (ρ = 0.156), maintenance/optimization of drainage (ρ = 0.113), interventions associated with complementary examinations of the medical diagnosis (ρ = 0.156), administration of drugs or other therapeutic components (ρ = 0.127), cardiovascular support (ρ = 0.114) and other specific highly differentiated interventions carried out in the unit of intensive care (ρ = 0.111) also increases.

The two statistically significant linear associations with the *length of professional experience* can be described as follows: negative with *situations of persistence of biophysiological indicators incompatible with life* (ρ = −0.129) and positive with *specific interventions carried out outside the ICU* (ρ = 0.105), which suggests that as the *length of professional experience* increases, the perception of futility in the above situations decreases, and vice versa, and the perception of futility in specific interventions increases.

A positive linear association was found between the variable *length of professional experience in adult intensive care* and *mobilization/positioning* (ρ = 0.098), suggesting that as professional experience in these settings increases (or decreases), the perception of futility of *mobilization/positioning* also increases (or decreases). Statistical analysis revealed three positive linear associations between the *professional category* and *monitoring* (ρ = 0.165), *hygiene care* (ρ = 0.119), and *dressings* (ρ = 0.157), suggesting that as the *professional category* of nurse increases (or decreases), the perception of futility of these interventions increases (or decreases).

Four positive linear associations were identified between *ICU level* and *situations of high irreversibility* (ρ = 0.142) and three interventions, *administration of drugs or other therapeutic components* (ρ = 0.121), *renal replacement therapy* (ρ = 0.137), and *neurological support* (ρ = 0.099). These associations suggest that with increasing (or decreasing) *ICU level*, the perception of futility also increases (or decreases) in *situations of high irreversibility* and *administration of drugs or other therapeutic components*, *renal replacement therapy,* and *neurological support*.

Given the polytomous nominal nature of the variables of *nursing specialty* and *ICU type*, it was only possible to assess the intensity of the association between the variables. Two identical moderate associations were identified between the *nursing specialty* variable and *situations of persistence of biophysiological indicators incompatible with life* (ϕc = 0.202) and *situations of high irreversibility* (ϕc = 0.202). With the *ICU type*, two weak associations, although statistically significant, were identified with *situations of persistence of biophysiological indicators incompatible with life* (ϕc = 0.149) and *situations of high irreversibility* (ϕc = 0.193).

Among the statistically significant associations found, it is important to mention the positive linear associations found between *educational level*, *professional category,* and *nursing specialty* with the variables related to the perception of futility in the situations analyzed and the perception of futility of nursing interventions, suggesting that as *educational level*, *professional category,* and *nursing specialty* increase (or decrease), the perception of futility also increases (or decreases).

*Educational level*, *professional category,* and *nursing specialty* share knowledge and competence, attributes directly related to wisdom and expertise. Regarding these concepts, it is worth recalling Benner (2001) and his perspective on the final stage of the skill acquisition model, in which nurses have a level of knowledge that allows them to understand problems intuitively and immediately, and to anticipate future intervention needs without the need for support obtained solely analytically.^31^ The results seem to suggest an association between greater perceptions of futility in *monitoring* and *educational level*, *professional category,* and/or *nursing specialty*. Similar results were reported by Mohammadi and Roshanzadeh (2015) and Nazari et al. (2022), who reported a greater perception of this phenomenon associated with education level and years of professional experience.^22,32^ Moaddaby et al. (2021) even showed a trend towards a higher perception of futility in nurses with a master’s degree.^23^

The statistical inference method used, bootstrapping, allowed us to estimate ranges within which the mean and median values of the dependent variables can be identified 95% of the time in 95% of the replicates extracted.^15,16^ The results of the confidence intervals for the mean confirm intervals with narrow amplitudes for the variables related to specific situations and nursing interventions.

Analysis of the results obtained by calculating the median confidence intervals for the two specific situations shows that the lower and upper confidence intervals are the same. For nursing interventions, the lower and upper confidence intervals are equal to the median of twelve interventions. However, for the other eight interventions, the interval is very narrow.

Traditionally, ICUs are equipped with resources that allow them to provide care according to current knowledge. However, because nurses in these units are often confronted with critically ill patients, irreversible situations, and high mortality and morbidity rates, these environments are highly stressful and systematically expose nurses to moral crises.^33–35^ The promotion of futile nursing interventions can catalyze nurses’ moral suffering and negatively affect the quality of care provided. Although the authors believe these findings should be interpreted cautiously, the statistical inference results lead them to consider the possibility that nurses in level II and III ICUs may perceive the frequent implementation of futile nursing interventions in critically ill patients.

### Study strengths and limitations

The main strength of this study is the meticulous adherence to a rigorous methodological process, including the design of the questionnaire and its subsequent validation, as well as a comprehensive statistical analysis. This analysis was conducted under a statistician’s supervision, ensuring the results’ reliability.

The lack of quantitative studies analyzing futile nursing interventions in adult ICU makes it challenging to compare the findings with other evidence, representing the authors’ primary identified limitation of this research. The second limitation is related to the inference method, the bootstrapping. Despite being a valid strategy that produces robust estimates for the mean, an approach supported by several authors,^16,17^ it is challenging to disregard the fact that the traditional estimation method employed in numerous studies dealing with ordinal variables is the median and mode.^18^ However, in particular research domains, such as the social sciences, calculating the mean in ordinal variables is a prevalent strategy.^19^

## Conclusion

The findings of this research indicated a balance in the nurses’ perceptions of the futility of their interventions in the specific situations observed. The data obtained supports the assertion that nurses perceive the futility of interdependent interventions more frequently than autonomous interventions.

This research has significant practical implications, as it contributes to the scientific knowledge of nursing regarding futile nursing interventions adult ICU. The findings allow nurses to reflect on their implementation in advance, which may lead to more effective and efficient care.

The next phase of this research will entail assessing the construct and criterion validity of the instrument developed for this study. The exploratory and confirmatory factor analysis techniques made it possible to contribute to a scientifically tested and validated instrument to facilitate new studies on therapeutic futility in nursing.

The authors recommend undertaking further quantitative studies to analyze the futility of nursing interventions implemented in critically ill patients admitted to adult ICU.

## Data Availability

The data supporting the findings of this study is available from the corresponding author upon justified request.*

## Notes

1. *Correlation is significant at the 0.05 level (2 tailed).

2. **Correlation is significant at the 0.01 level (2 tailed).
